# Inflammatory response of human dental pulp to at-home and in-office tooth bleaching

**DOI:** 10.1590/1678-775720160137

**Published:** 2016

**Authors:** Maysa Magalhães Vaz, Lawrence Gonzaga Lopes, Paula Carvalho Cardoso, João Batista de Souza, Aline Carvalho Batista, Nádia Lago Costa, Érica Miranda Torres, Carlos Estrela

**Affiliations:** 1 Universidade Federal de Goiás, Faculdade de Odontologia, Goiânia, GO, Brasil.; 2 Universidade Federal de Goiás, Faculdade de Odontologia, Departamento de Ciências Estomatológicas, Goiânia, GO, Brasil.

**Keywords:** Tooth bleaching, Inflammation, Dental pulp, Microscopy, Immunohistochemistry

## Abstract

Tooth bleaching is a technique of choice to obtain a harmonious smile, but bleaching agents may damage the dental pulp.

**Objective:**

This study evaluated the inflammatory responses of human dental pulp after the use of two bleaching techniques.

**Material and Methods:**

Pulp samples were collected from human third molars extracted for orthodontic reasons and divided into three groups: control - no tooth bleaching (CG) (n=7); at-home bleaching with 15% carbamide peroxide (AH) (n = 10), and in-office bleaching with 38% hydrogen peroxide (IO) (n=12). Pulps were removed and stained with hematoxylin-eosin for microscopic analysis of inflammation intensity, collagen degradation, and pulp tissue organization. Immunohistochemistry was used to detect mast cells (tryptase^+^), blood vessels (CD31^+^), and macrophages (CD68^+^). Chi-square, Kruskal-Wallis, and Mann Whitney tests were used for statistical analysis. The level of significance was set at p<.05.

**Results:**

The inflammation intensity and the number of macrophages were significantly greater in IO than in AH and CG (p<0.05). The results of CD31^+^ (blood vessels per mm^2^) were similar in CG (61.39±20.03), AH (52.29±27.62), and IO (57.43±8.69) groups (p>0.05). No mast cells were found in the pulp samples analyzed.

**Conclusion:**

In-office bleaching with 38% hydrogen peroxide resulted in more intense inflammation, higher macrophages migration, and greater pulp damage then at-home bleaching with 15% carbamide peroxide, however, these bleaching techniques did not induce migration of mast cells and increased the number of blood vessels.

## INTRODUCTION

Tooth bleaching has been widely used to correct tooth discolorations and to produce a harmonious smile^13^. Techniques to improve tooth color include the use of whitening toothpastes, professional cleaning by scaling and polishing to remove stain and tartar, tooth bleaching, microabrasion of enamel with abrasives and acid, and placement of crowns and veneers. Tooth bleaching, in particular, is an effective and conservative technique to treat discolored teeth^11-13^.

Several techniques and approaches available for vital tooth bleaching^11-13^ vary in type of bleaching agent, its concentration, time of application, product presentation, application mode, and light activation^11-13^. Main differences between techniques consist in bleaching agent concentration and application time^11,12^. Three bleaching approaches have been used: at-home bleaching, in-office bleaching, and a combination of both techniques^2,11,13^. For at-home bleaching, studies recommend the use of a low concentration of bleaching agent (10-16% carbamide peroxide) applied for at least 16 days^2,11,13^. In power bleaching, or in-office bleaching, hydrogen peroxide (25-38%) is applied for shorter time periods^2^.

As oxygen diffuses through enamel and dentin, it may affect the pulp^11,12,18,21,28^ and result in tooth sensibility, an adverse effect of toxic components^2,3,12,15,27^. The low molecular weight of hydrogen peroxide promotes tooth bleaching, but also the release of inflammatory mediators into the pulp, which may damage pulp cells^3,12,26,27^. The rate of hydrogen peroxide penetration differs between bleaching agents and depends on their concentration and time of application as well as on the presence or absence of restorations^3,17,21^.

The inflammatory response caused by chemical aggressors involves non-specific events that include vascular-exudative phenomena and infiltration of inflammatory cells such as mast cells and macrophages^1^. These cells play an important role in pulp defense, but they also participate in the degradation of the extracellular matrix, neovascularization, cell recruitment and repair^20,23,29^. Nevertheless, mast cells are responsible for the secretion of histamine, a vasodilator that participates in the early inflammatory events against aggression^1,7,16^.

Several researchers evaluated the bleaching agents damage in dental pulps of animal models and *in vitro* studies^3,8,17,22^. However, few studies aimed to evaluate the effect of bleaching agents on human pulps^9,13,22,24^. Hence, pulp inflammatory response should be better understood before a bleaching technique is clinically used. This study used immunochemistry to evaluate some inflammatory events and cells involved in the human pulp response to at-home and in-office tooth bleaching. The null hypothesis was that there would be no pulp changes after at-home and in-office tooth bleaching.

## MATERIAL AND METHODS

### Sample selection

Twenty-nine human pulps were collected from caries-free third molars extracted for orthodontic reasons at the Dentistry Clinic of the School of Dentistry, Federal University of Goiás, Brazil. Patient's mean age was 23.5 years (ranging from 17 to 30 years). The clinical histories of all patients were reviewed to ensure that the experimental group included only healthy patients. All teeth extracted were fully erupted and healthy (no cracks, fractures, caries, or restorations). Pulp vitality was confirmed in all teeth using 1,1,1,2-tetrafluoroethane (Endo-Ice-Hygenic Corp; Akron, OH, USA). This study was approved (protocol #281/11) by the Research Ethics Committee of the institution where it was conducted, and all participants signed an informed consent form.

### Study design

Pulp samples were randomly divided into three groups according to the bleaching technique used. Molars were extracted seven days after tooth bleaching. No tooth bleaching was used in the control group (CG) (n=7). In the second group, at-home bleaching with 15% carbamide peroxide (AH, n=10) (Opalescence PF 15- Ultradent Products; South Jordan, UT, USA) was applied for 16 days, 2 hours a day. In the third group, in-office bleaching with 38% hydrogen peroxide group (IO, n=12) (Opalescence Xtra Boost-Ultradent Products, South Jordan, UT, USA) was applied for 45 minutes in each of three visits. All tooth bleaching systems were used following manufacturer's instructions.

Immediately after tooth extraction, the apical third of the roots was removed using a #2136 diamond point (KG Sorensen Ind. Com. Ltda; São Paulo, SP, Brazil) and a water-cooled air-driven handpiece. The samples were preserved in 10% buffered formalin (pH 7.4) for 48 hours. After that, perpendicular grooves were made along the long axis of the teeth with a #4219 high-speed diamond bur (KG Sorensen Ind. Com. Ltda.; São Paulo, SP, Brazil), and the fragments were removed with a bone chisel, in such a way that the entire dental pulp was removed. Pulp tissues were again preserved in 10% buffered formalin for microscopic analysis.

### Microscopic analysis

Specimens were embedded in paraffin and subsequently sectioned using a microtome (RM2165- Leica Microsystem Inc.; Buffalo Grove, IL, USA). One hematoxylin-eosin (H&E) stained 5-μm section from each sample was used for the microscopic evaluation of inflammation intensity, collagen degradation, and pulp tissue organization. Microscopic analysis was performed under trinocular microscopy (Axiostar Plus- Carl Zeiss; Jena, Turingia, Germany).

### Immunohistochemistry

Immunohistochemistry was performed using a primary mouse monoclonal antibodies: anti-human tryptase (mast cell) (M7052- DAKO; Glostrup, Copenhagen, Denmark), anti-human CD31 (endothelial cell) (clone 1A10- Novocastra; Newcastle, UK, USA), and anti-human CD68 (macrophage) (clone KP1- Novocastra; Newcastle, UK, USA).

Paraffin-embedded tissues were sectioned to obtain 3-um serial sections using a microtome (RM2165- Leica Microsystem Inc.; Buffalo Grove, IL, USA) and mounted on glass slides coated with 2% (3-aminopropyl)triethylsilane (Sigma Chemicals; St Louis, MO, USA). The sections were deparaffinized in xylene, rehydrated in decreasing concentrations of alcohol, and then incubated with 3% hydrogen peroxide for 40 minutes. For antigen retrieval, the sections were immersed in a citrate buffer (pH 6.0) for 20 minutes at 95°C, except for the tryptase antibody. Afterwards, the sections were incubated for 20 minutes with 3% normal goat serum at room temperature. Next, the slides were incubated at 4°C overnight with the primary antibody in a humidified chamber. Antibody dilutions were 1:2000 for tryptase, 1:200 for CD31, and 1:1000 for CD68. After washing with phosphate-buffered saline, the sections were prepared using a labeled streptavidin-biotin (LSAB) kit (K0492- DAKO; Glostrup, Copenhagen, Denmark) and then incubated with 3,3'-diaminobenzidine (K3468-DAKO; Glostrup, Copenhagen, Denmark) for 2-5 minutes at room temperature. The sections were stained with Mayer's hematoxylin and covered. Negative controls were obtained by omitting the primary antibodies, which were replaced with 1% PBS-BSA and non-immune mouse (X501-1-DAKO; Glostrup, Copenhagen, Denmark) serum. Periapical granulomas samples were used as positive controls.

### Semiquantitative, quantitative, and statistical analysis

For microscopic analysis, histological features (inflammation intensity, collagen degradation, and pulp tissue organization) were scored as described below.

The gradation of the inflammation was performed as described previously by Bruno, et al.^20^ (2010). It was determined in 10 representative microscopic high-power fields (400x magnification) using an integration graticule (Carl Zeiss; Jena, Turingia, Germany). The specimens were scored according to inflammation intensity: 0, when most fields (>7) had no inflammatory cells; 1, when most fields (>7) had <35% of the integration graticule filled with inflammatory cells; and 2, when most fields (>7) had more than 35% of the integration graticule filled with inflammatory cells.

The evaluation criteria for pulp tissue organization were adapted from Soares, et al.^14^ (2014). Pulp tissue organization was scored as follows: 0, normal tissue, with preserved odontoblastic layer and central pulp; 1, disorganized odontoblastic layer, but normal central pulp; 2, total disorganization of pulp tissue morphology, but no areas of necrosis; 3, pulp necrosis. Collagen was classified as preserved (0) or degraded (1).

In immunohistochemistry, the number of cells or vessels/mm^2^ of mast cells tryptase^+^, blood vessels CD31^+^, and macrophage CD68^+^ were determined using an integration graticule (12.5×, 4740680000000- Carl Zeiss; Jena, Turingia, Germany). Only blood vessels with visible vascular lumens were counted. Cells and blood vessels were identified in 10 representative and consecutive microscopic high-power fields (×400) and, at this magnification, each field of the integration graticule had an area of 0.0961 mm^2^. Descriptive analyses were expressed as mean ± standard deviation (SD) of n observations, *per* mm^2^.

Data were analyzed using a chi-square test for microscopic findings and the Wilcoxon test followed by the Mann-Whitney test for immunohistochemistry findings. Statistical analyses were performed using the SPSS 21.0 for Windows, and the level of significance was set at p<.05.

## RESULTS

**Figure 1:**
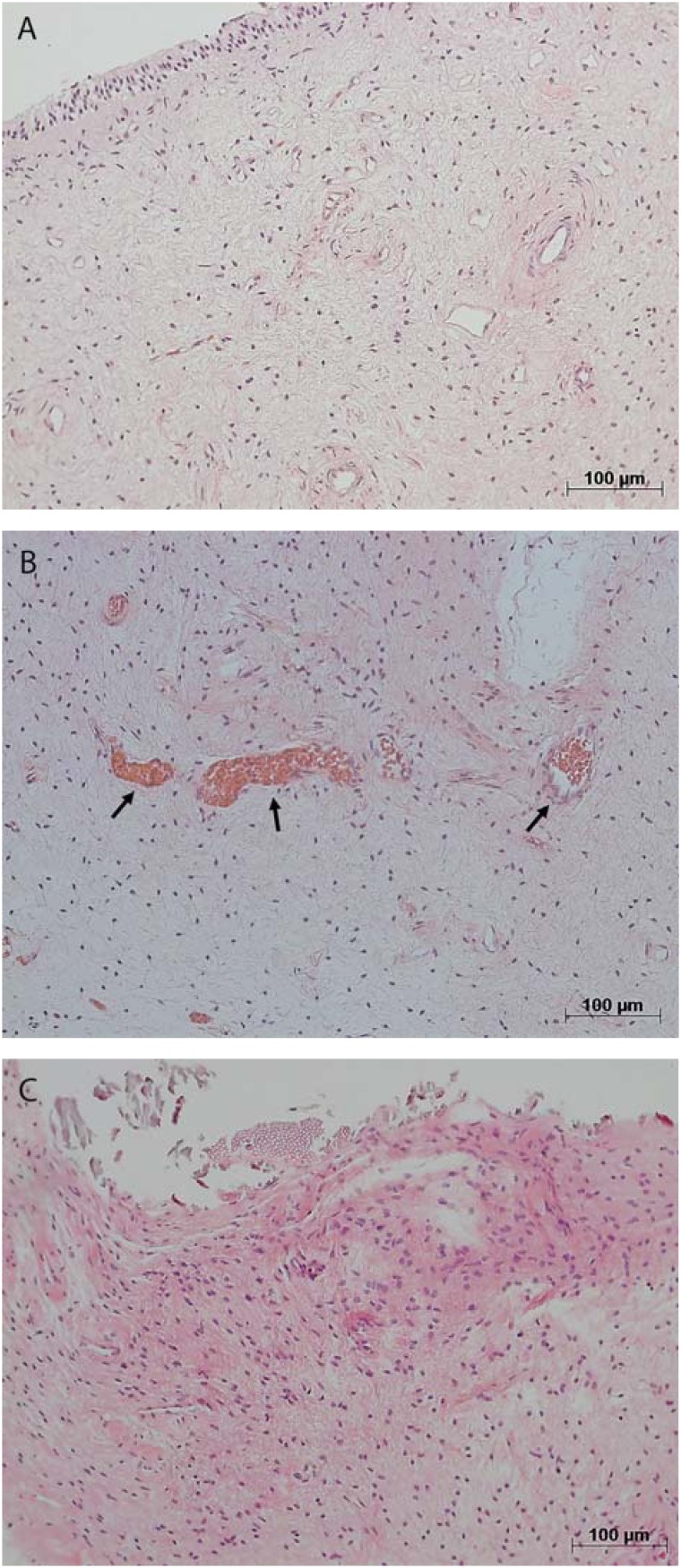
Representative images showing dental pulp with absence of inflammatory infiltrate (score 0) and preserved blood vessels in control group (A); absent inflammatory infiltrate (score 0) and dilated and congested blood vessels (arrows) in at-home bleaching group (B); and mild inflammatory infiltrate (score 1) in in-office bleaching group (C). Hematoxylin & eosin staining, original magnifications 100X;A-C

**Figure 2:**
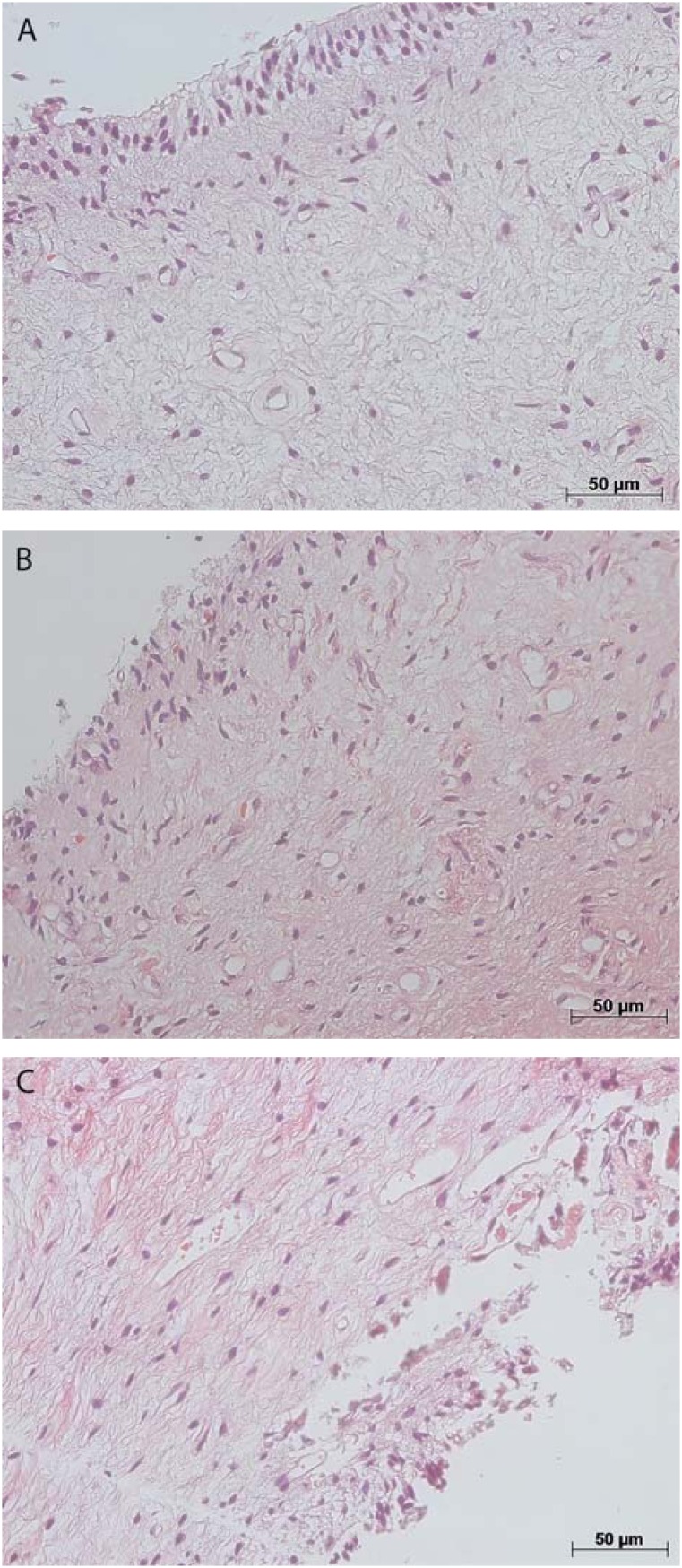
Photomicroscopy illustrating preserved collagen (score 0) and well-organized pulp tissue (score 0) in control group (A); preserved collagen (score 0) and disorganized pulp tissue (score 1) in at-home bleaching group (B); and degraded collagen (score 1) and disorganized pulp tissue (score 2) in in-office bleaching group (C). Hematoxylin & eosin staining, original magnifications 200X; A-C

The microscopic analysis of the dental pulp samples in the CG showed that collagen was preserved (score 0) in 86%, pulp tissue was well organized (score 0) in 71%, and inflammatory infiltrate was absent (score 0) in 100%. There were small-caliber blood vessels in the pulp tissue (Figure 1A and 2A). In AH, collagen was preserved (score 0) and no inflammatory infiltrate was found (score 0) in 80% of the samples, whereas pulp tissue was disorganized (scores 1 and 2) in 60% of cases. There were a large number of congested small-caliber blood vessels (Figure 1B and 2B). In contrast, in the pulp samples in IO, the inflammation intensity was mild (score 1) or intense (score 2) in 75% of the cases, collagen was degraded (score 1) in 58%, and pulp tissue was disorganized (scores 1 and 2) in 83%. There were congested blood vessels of a larger caliber in the pulp samples (Figures 1C and 2C).

The intensity of inflammation was significantly greater in IO than in AH and in the CG (p=0.038 and p=0.005, respectively). There were also differences in collagen degradation and pulp tissue organization between the samples in IO, since collagen was degraded and pulp tissue was disorganized, but this difference was not statistically significant (p>.05). Table 1 shows the microscopic features of the samples.

**Table 1:** Histopathologic features according to study groups (absolute and relative frequencies)

Histopathologic features	Group	Scores			
		0	1	2	3
Intensity of inflammation	CG	7 (100%)	0 (0%)	0 (0%)	--
	AH	8 (80%)	2 (20%)	0 (0%)	--
	IO	3 (25%)	8 (67%)	1 (8%)	--
					
Collagen degradation	CG	6 (86%)	1 (14%)	--	--
	AH	8 (80%)	2 (20%)	--	--
	IO	5 (42%)	7 (58%)	--	--
					
Pulp tissue organization	CG	5 (71%)	2 (29%)	0 (0%)	0 (0%)
	AH	4 (40%)	5 (50%)	1 (10%)	0 (0%)
	IO	2 (17%)	6 (50%)	4 (33%)	0 (0%)

Groups: CG (n=7): control group, no bleaching; AH (n=10): at-home bleaching; IO (n=12): in-office bleaching

* Represents significant difference when compared with AH and CG (Qui Square Test; p<0.05)

**Figure 3:**
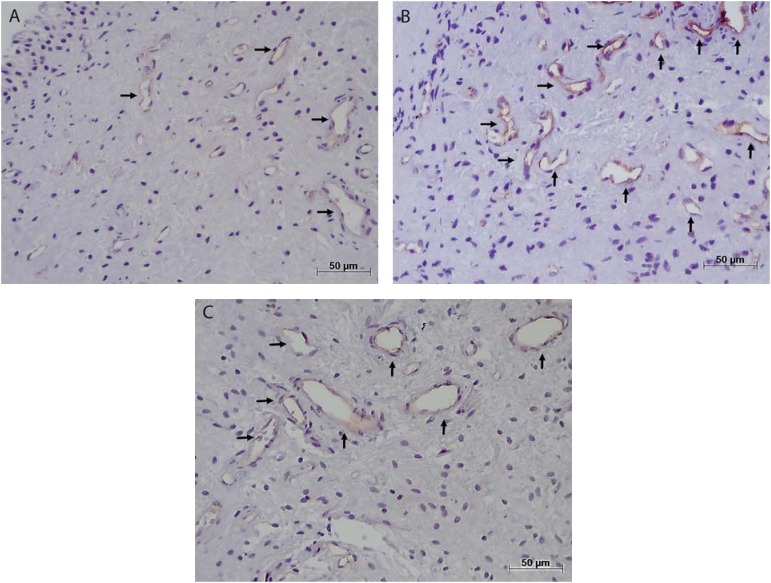
Blood vessels (CD31+) (arrows) in control group (A), at-home bleaching group (B), and in-office bleaching group 2 (C). Immunohistochemical staining; original magnifications 200×; A-C

**Figure 4:**
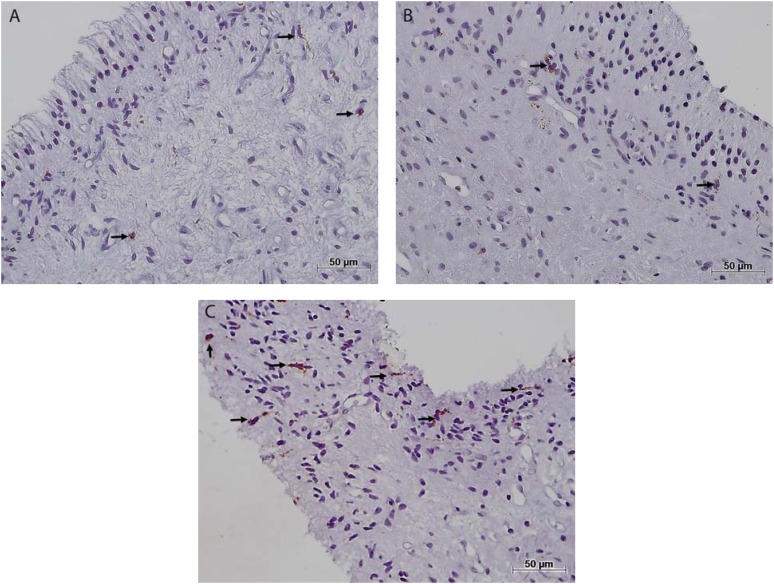
Macrophages (CD68+) (arrows) in the dental pulp tissue of control group (A), at-home bleaching group (B), and in-office bleaching group (C); A and B images show low numbers of these cells in control group and in at-home bleaching group; C image Illustrates the high number of macrophages in in-office bleaching group. Immunohistochemical staining; original magnifications 200×; A-C

The immunohistochemical analysis showed that CD68+ cells had a brown staining pattern in membranes and cytoplasm (Figure 4). In addition, CD31 was found in endothelial cells of blood vessels (Figure 3). No mast cells (tryptase^+^) were found in the pulp samples analyzed.

The number of CD68^+^ cells *per* mm^2^ was significantly higher in IO (38.14±19.73) than in AH (16.99±8.91) and in CG (13.58±10.44) (Mann-Whitney, p=0.01 and p=0.028). Although the number of CD68^+^ cells was higher in AH samples than in CG, this difference was not statistically significant (Mann-Whitney, p=0.46). The number of blood vessels CD31^+^ *per* mm^2^ was similar in the three groups (CG, 61.39±20.03; AH, 52.29±27.62; IO, 57.43±8.69) (Mann–Whitney, p>.05) (Table 2). There was no relation between the number of macrophages and of blood vessel in IO, AH, and control groups (p>0.05).

**Table 2:** Densities (mm^2^) of macrophages (CD68^+^) and blood vessels (CD31^+^) in control group, no bleaching technique performed (CG); home bleaching technique (AH); and in-office bleaching technique (IO)

	Means ± SD		
	CG (n=7)	AH (n=10)	IO (n=12)
Macrophages (CD68+)	13.58 ± 10.44	16.99 ± 8.91	38.14 ± 19.73*
Blood vessels (CD31+)	61.39 ± 20.03	52.29 ± 27.62	57.43 ± 8.69

The Mann-Whitney test was used to compare groups at a 0.05 significance level.

* Represents significant differences when compared with CG and AH groups.

## DISCUSSION

Bleaching with 15% carbamide peroxide (at-home bleaching) or 38% hydrogen peroxide (in-office bleaching) resulted in pulp inflammation characterized by an increase in vascular dilation, in the number of macrophages, and inflammatory intensity response. However, this response seemed to be more intense when in-office bleaching was used. Thus, the null hypothesis was rejected.

Dental materials, including bleaching agents^11,12,18,21,24,26-28^, may cause cell damage in human dental pulp tissues^4,19,25^. The results of studies that examine the effects of bleaching agents on the pulp-dentin complex may be influenced by several factors such as animal model adopted, bleaching agent and its concentration, group of teeth that undergo bleaching, and duration and number of applications. Bowles and Thompson^5^ (1986) studied the effects of heat and hydrogen peroxide on pulp enzymes and found that both agents tested had a destructive effect, since they inactivated those enzymes and consequently disrupted normal cell activities. Bowles and Ugwuneri^6^ (1987) found that the use of high temperature increases the penetration of hydrogen peroxide into the dental pulp. Cintra, et al.^11^ (2013) evaluated the effect of number of bleaching sessions on the pulp tissue of mice and found that all pulp samples had significant changes induced by bleaching, and that changes increased with number of sessions.

In our study, concentration and bleaching technique affected pulp response. Inflammation with increased macrophage migration was more intense in pulps in the AH group that in those in the IO group. The analysis of inflammation intensity (Table 1) showed that the IO group had the highest number of pulp samples with moderate or severe inflammation, whereas most (80%) of the samples in the AH group had no inflammatory infiltrate, and no samples in the CG had any inflammatory infiltrate. The increase in inflammatory infiltrate from the CG to the IO group is shown in Figure 1.

Several factors may contribute to pulp inflammation during bleaching such as bleaching agent concentration, application time, and tooth group. In a study on bleaching agent concentration and application time, Soares, et al.^26^ (2014) evaluated the efficacy and toxicity of different bleaching techniques in pulp cells. They tested different agent concentrations (17.5% or 35%) and application times (1×5 min; 1×15 min; 3×15 min) using enamel and dentin discs placed on odontoblast-like cells (MDPC-23). The reduction in bleaching time to 5 minutes when hydrogen peroxide was used at 35% or a reduction of 17.5% in concentration for any period of time produced gradual changes in tooth color and reduced toxicity to pulp cells.

Camargo, et al.^8^ (2007) compared the presence of hydrogen peroxide in the pulp chamber of human and bovine teeth. In their analysis according to tooth thickness, thinner human teeth had higher amounts of hydrogen peroxide in the pulp chamber. Costa, et al.^12^ (2010) found irreversible pulp changes and areas of necrosis in human incisors, but not in premolars that underwent in-office tooth bleaching. Robertson and Melfi^24^ (1980) and Kina, et al.^22^ (2010) found no pulp changes in human premolars treated with in-office bleaching. The analysis of our results should consider that human molars were used.

This study investigated, for the first time, the presence of mast cells (tryptase^+^), macrophages (CD68^+^), and blood vessels (CD31^+^) in human dental pulp after the use of two bleaching techniques. The evaluation of the mast cell density, macrophages, and endothelial cells at the site of tissue injury, by a specific technique, such as immunohistochemistry, allows investigating cells involved in diverse events of the innate immune response, such as vasodilation, angiogenesis, and extracellular matrix degradation, usually present in tissue aggression caused by chemical agents.

The present study demonstrated that there were no mast cells in human dental pulp in experimental and control groups. Mast cells are present in all connective tissues of the body, including those in the oral cavity, but there is still controversy about whether they are found in normal and inflamed pulp^7,14,16^. Freitas, et al.^16^ (2007) used immunohistochemistry to evaluate the presence of mast cells in 40 samples of human dental pulps of permanent teeth in different clinical circumstances: unerupted teeth, partially erupted teeth without dental caries, erupted teeth without dental caries, erupted teeth with shallow carious dentin, and teeth with hyperplastic pulpitis (pulp polyp). Mast cells were found only in the samples of hyperplastic pulpitis. Dockrill^14^ (1961) also found mast cells only in pulp polyps. Similarly, our study did not find mast cells (tryptase^+^ cells) in dental pulps. These findings suggest that other mediators not secreted by mast cells, such as substance P (SP)^9^, may be responsible for the regulation of blood flow and vascular permeability during pulp inflammation. Substance P has been found in the sensory nerve fibers of the pulp, both in the center, next to blood vessels, and in the periphery^10^. Neuropeptides are key in the generation of neurogenic inflammation, and their levels are increased during caries, inflammation, and occlusal trauma as well as after cavity preparation, application of dentin bonding agents, and tooth bleaching^6^.

This study found a higher density of macrophages, collagen degradation, and infiltrate inflammatory in the pulps that underwent in-office bleaching with 38% hydrogen peroxide (applied for 45 minutes in each of three visits), which suggests that this bleaching protocol may cause more aggressive damage in dental pulp than at-home bleaching with 15% carbamide peroxide (applied for 16 days, 2 hours a day) (Table 1 and 2; Figure 4). Macrophages have many functions in inflammatory response, including degradation of the extracellular matrix by producing a large number of matrix metalloproteinases (MMPs-1, 3, and 9)^20,23,29^ and also chemoattractant that recruit additional leukocyte and pro-inflammatory cytokines, including Interleukin-1, 6, 12, and tumor necrosis factor alpha (TNF-α)^23^. Moreover, the macrophages are involved in neovascularization, pulp repair, and fibroblast proliferation, by expression of cytokines and growth factors, such as vascular endothelial growth factors (VEGF), fibroblast growth factors (FGF), transforming growth factor beta (TGF-β), and insulin-like growth factor (IGF)^20,23,29^. Nevertheless, in the present study there was no relation between the number of macrophages and of blood vessel in IO and AH groups. Taken together, these findings suggest that when facing a chemical injury, macrophages may more intensely participate of collagen degradation (by metalloproteinase production) and of proinflammatory events than the vascular neoformation and tissue repair events. However, to confirm this hypothesis, clinical studies on the function of macrophages in chemical injury with bleaching agents should be performed.

This study found no differences in pulp tissue organization between groups. The mechanical method of dental pulp removal used in this study might have disorganized the odontoblastic layer of all pulps and, therefore, affect the evaluation of this criterion. This is a limitation of this study, and future studies using mostly the same method but replacing mechanical removal with demineralization should contribute to the clarification of this question.

The use of third molars in this study might be a limitation, but it may contribute to the understanding of the pulp inflammatory response in this group of teeth, in which the structure of the enamel-dentin-pulp complex is different from that found in other groups. However, the thickness of the enamel-dentin of these teeth may be associated with the type of pulp response found for the bleaching protocols under evaluation. In teeth with a thinner enamel-dentin complex, as well as in samples with cracks, cervical lesions or structural defects, inflammation may be more intense. For the tooth group evaluated here, results suggest that in-office bleaching in clinical practice may lead to damages.

Teeth were extracted seven days after bleaching in this study, and inflammation intensity might have been different if teeth had been extracted after a longer time. Repair might have happened earlier, and a different response pattern might be found. Further clinical trials should include the extraction of teeth at different periods after the completion of tooth bleaching.

The clinical relevance of this study lies in the fact that findings suggest that bleaching techniques should be carefully selected and that this treatment should be selectively indicated, since mild to moderate short-term changes were found in third molars that underwent in-office bleaching.

In summary, in-office bleaching of molars with 38% hydrogen peroxide (applied for 45 minutes in each of three visits) caused higher macrophages migration, more intense inflammation, and greater pulp damage than at-home bleaching with 15% carbamide peroxide (applied for 16 days, 2 hours a day). The number of blood vessels was similar in all groups, regardless of the tooth bleaching technique employed. Mast cells were not found in pulp tissue and were probably not associated with inflammation in healthy teeth submitted to chemical aggression.
